# Polymorphism rs11085226 in the Gene Encoding Polypyrimidine Tract-Binding Protein 1 Negatively Affects Glucose-Stimulated Insulin Secretion

**DOI:** 10.1371/journal.pone.0046154

**Published:** 2012-10-15

**Authors:** Martin Heni, Caroline Ketterer, Robert Wagner, Katarzyna Linder, Anja Böhm, Silke A. Herzberg-Schäfer, Fausto Machicao, Klaus-Peter Knoch, Andreas Fritsche, Harald Staiger, Hans-Ulrich Häring, Michele Solimena

**Affiliations:** 1 Division of Endocrinology, Diabetology, Angiology, Nephrology and Clinical Chemistry, Department of Internal Medicine, Eberhard Karls University Tübingen, Tübingen, Germany; 2 Institute for Diabetes Research and Metabolic Diseases of the Helmholtz Center Munich at the University of Tübingen, Partner in the German Center for Diabetes Research (DZD), Tübingen, Germany; 3 Department of Internal Medicine, Nutritional and Preventive Medicine, Eberhard Karls University Tübingen, Tübingen, Germany; 4 Molecular Diabetology, Paul Langerhans Institute Dresden, University Hospital and Medical School Carl Gustav Carus, Dresden University of Technology, Partner in the German Center for Diabetes Research (DZD), Dresden, Germany; University of Bremen, Germany

## Abstract

**Objective:**

Polypyrimidine tract-binding protein 1 (PTBP1) promotes stability and translation of mRNAs coding for insulin secretion granule proteins and thereby plays a role in β-cells function. We studied whether common genetic variations within the *PTBP1* locus influence insulin secretion, and/or proinsulin conversion.

**Methods:**

We genotyped 1,502 healthy German subjects for four tagging single nucleotide polymorphisms (SNPs) within the *PTBP1* locus (rs351974, rs11085226, rs736926, and rs123698) covering 100% of genetic variation with an r^2^≥0.8. The subjects were metabolically characterized by an oral glucose tolerance test with insulin, proinsulin, and C-peptide measurements. A subgroup of 320 subjects also underwent an IVGTT.

**Results:**

*PTBP1* SNP rs11085226 was nominally associated with lower insulinogenic index and lower cleared insulin response in the OGTT (p≤0.04). The other tested SNPs did not show any association with the analyzed OGTT-derived secretion parameters. In the IVGTT subgroup, SNP rs11085226 was accordingly associated with lower insulin levels within the first ten minutes following glucose injection (p = 0.0103). Furthermore, SNP rs351974 was associated with insulin levels in the IVGTT (p = 0.0108). Upon interrogation of MAGIC HOMA-B data, our rs11085226 result was replicated (MAGIC p = 0.018), but the rs351974 was not.

**Conclusions:**

We conclude that common genetic variation in *PTBP1* influences glucose-stimulated insulin secretion. This underlines the importance of PTBP1 for beta cell function *in vivo*.

## Introduction

Glucose stimulation of pancreatic β-cells induces the extracellular release of insulin by inducing the fusion of insulin secretory granules with the plasma membrane [1]. This process is additionally enhanced by incretins (e.g. glucagon-like peptide 1 [GLP-1]) via the second messenger cyclic adenosine monophosphate (cAMP) [2]. In addition to insulin release, glucose rapidly up-regulates the production of new insulin secretory granules. This is due to the enhanced synthesis of most insulin secretory granule components [3]–[5], like pre-proinsulin, the prohormoneconvertasesPCSK1 and PCSK2 [5]–[7], chromogranins [8] and the islet cell antigen (ICA) 512/IA-2 [9]. This rapid induction of insulin secretory granule biogenesis is mainly caused by post-transcriptional mechanisms [7], [8], [10]–[12].

Polypyrimidine tract-binding protein 1 (PTBP1), formerly known as heterogeneous nuclear ribonucleoprotein I (hnRNP I), enhances mRNA stability and translation, in addition to regulating other processes [13]. For instance, PTBP1 mediates splicing repression in a long list of pre-mRNAs and is thereby important for the regulation of alternative splicing in a tissue-specific manner [14], [15]. In pancreatic β-cells and chromaffin cells, protein kinase A-induced phosphorylation of PTBP1 promotes its rapid shift from the nucleus into the cytoplasm [13], [16], [17]. Glucose stimulation of β-cells induces a similar nucleocytoplasmic translocation of PTBP1 [4], [13], [16]. By interacting with binding sites within the 3′- and 5′-UTRs (untranslated regions), cytosolic PTBP1, in turn, regulates mRNA localization, stability and the initiation of translation [14], [18]–[20]. Specifically, PTBP1 stabilizes the mRNA coding for insulin and other major insulin secretory granule components such as PCSK1, PCSK2, chromogranin A and ICA512 [4], [13], [16]. Accordingly, knockdown of PTBP1 in insulinoma INS-1 cells leads to the depletion of insulin secretory granule stores [4].

Impaired insulin secretion together with insulin resistance is a key factor in the pathogenesis of type 2 diabetes mellitus. Various mechanisms could be responsible for impairment of insulin secretion, including a decrease in the number of pancreatic β-cells due to apoptosis as well as impaired exocytosis or biogenesis of insulin secretory granules [21], [22]. Besides insulin transcription itself, the conversion of proinsulin into insulin and the cleavage of its C-terminal residues by carboxypeptidase E (CPE) are critical for insulin maturation [5], [7], [23], [24]. An increased proinsulin/insulin ratio is a characteristic finding at the onset of type 2 diabetes [25], [26]. Notably, PTBP1 promotes the translation of PCSK1 and PCSK2, but not those of CPE, whose translation is not regulated by glucose [4], [8], [13].

In view of these considerations, genetic variation in *PTBP1* might affect insulin secretion or maturation in humans. To test this hypothesis, we examined the association of common genetic variants (minor allele frequency [MAF]>5%) in *PTBP1* with both insulin secretion and proinsulin-to-insulin conversion.

## Methods

### Participants

The participants were selected from the ongoing Tübingen Family Study, which currently includes ∼2,000 individuals at increased risk of diabetes [27]. Individuals on medication affecting glucose metabolism were excluded. Inclusion of the participants in the present study was based on availability of: (i) DNA samples for genotyping (n = 1,750); and (ii) complete OGTT data (glucose, insulin, C-peptide and proinsulin levels available for all time points during the OGTT, n = 1,502).

The anthropometric characteristics of the study population are shown in Table 1. Most (69%) of these subjects had a family history of diabetes, i.e. at least one second-degree relative with type 2 diabetes mellitus. All participants were genotyped for the following SNPs: *PTBP1* rs351974, rs11085226, rs736926, and rs123698. Informed written consent was obtained from all participants and the local Ethics Committee (University of Tübingen) approved the protocol.

**Table 1:** Clinical characteristics of the study population.

Gender (female/male)	997/505
NGT/IFG/IGT/(IFG+IGT)	1095/151/146/110
Age (y)	39±13
BMI (kg/m^2^)	28.5±7.8
Waist circumference (cm)	93±17
Fasting glucose (mM)	5.09±0.54
Glucose, 120 min OGTT (mM)	6.26±1.65
Fasting insulin (pM)	62.4±52.4
Insulin, 30 min OGTT (pM)	430±460

Data are given as means ±SD. BMI – body mass index; IFG – impaired fasting glucose; IGT – impaired glucose tolerance; NGT – normal glucose tolerance; OGTT – oral glucose tolerance test.

### Selection of tagging SNPs and genotyping

**Figure 1:**
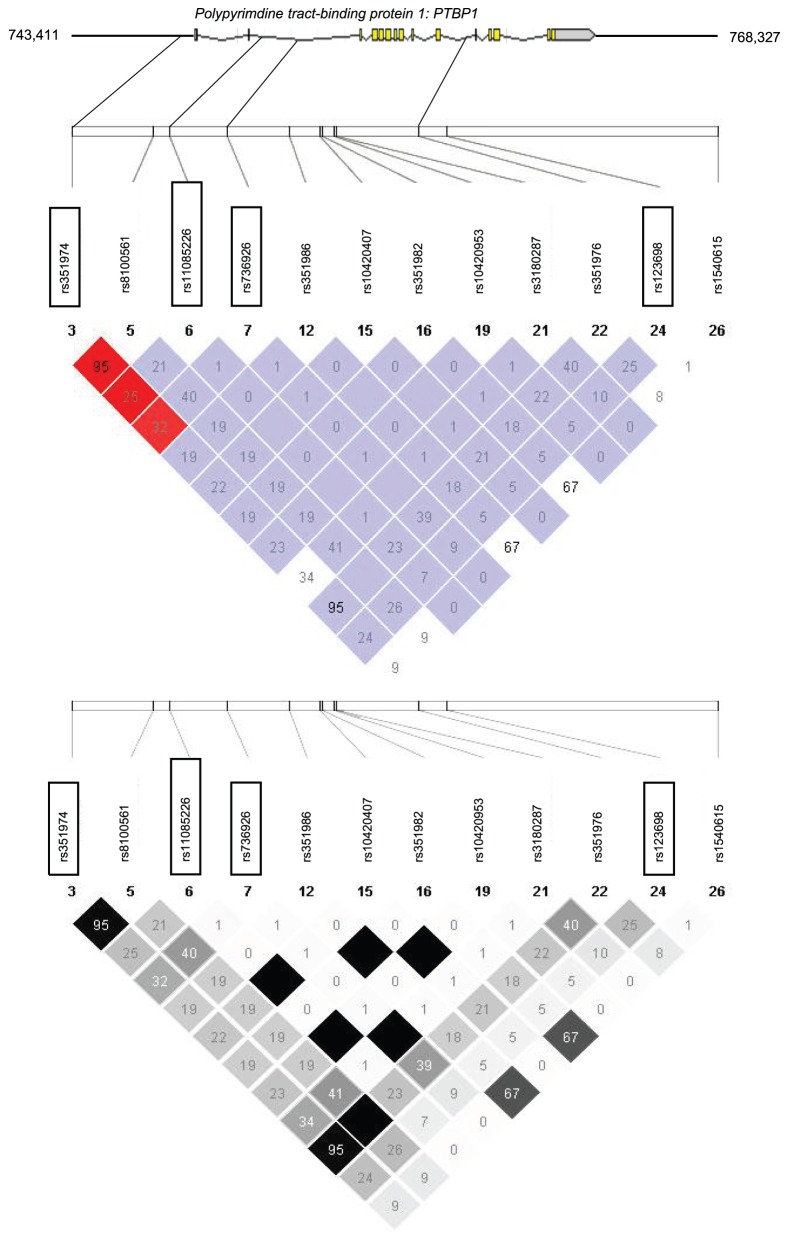
Genomic region of human chromosome 19p13.3 harboring the PTBP1 gene and HapMap linkage disequilibrium data of common (MAF>5%) informative SNPs within this locus. The PTBP1 gene consists of 15 exons and 14 introns. It spans 14.92 kb from nucleotide 748,411 to 763,327. The analyzed locus included 5 kb of both the 5′- and the 3′-flanking regions. The locations of the four tagging SNPs are marked by frames. The upper panel shows D′ values and the lower panel shows r^2^ values (black diamonds without values: r^2^ = 1.0).

Using the publically available phase II data of the International HapMap Project derived from a population of Utah residents with ancestry from Northern and Western Europe (release 24, November 2008, http://www.hapmap.org, [28]), we screened *in silico* the complete *PTBP1* gene (14.92 kb, 15 exons, 14 introns, located on human chromosome 19p13.3) and 5 kb of both the 3′ and 5′ flanking regions (Figure 1).

Twelve informative SNPs with minor allele frequency (MAF)>5% are located in this region. Their HapMap linkage disequilibrium data (D′ and r-squared values) are shown in Figure 1. Among these, we selected four tagging SNPs that cover 100% of the common genetic variation (MAF>5%) within this locus for genotyping: *PTBP1* rs351974 (A/C) is located in the 5′ flanking region of the gene, rs11085226 (A/G) and rs736926 (C/T) are located within intron 2, while rs123698 (C/G) is located in intron 10.

DNA from whole blood was isolated using a commercial DNA isolation kit (NucleoSpin, Macherey& Nagel, Düren, Germany). Genotyping was done using the TaqMan assay (Applied Biosystems, Forster City, CA, USA). The TaqMan genotyping reaction was amplified on a GeneAmp PCR system 7000 and fluorescence was detected on an ABI PRISM 7000 sequence detector (Applied Biosystems). The genotyping success rate for *PTBP1* rs351974 was 99.9%, for rs11085226 was 99.9%, for rs736926 was 99.8% and for rs123698 was 99.4%. Genotypes were verified in 50 randomly selected subjects by bidirectional sequencing, and both methods gave identical results.

### Oral glucose tolerance test (OGTT)

After an overnight fast of 10 h, subjects ingested a solution containing 75 g glucose at 08:00 hours. Venous blood samples were obtained at 0, 30, 60, 90, and 120 min and plasma glucose, insulin, C-peptide, and proinsulin concentrations were determined. Insulin sensitivity during the OGTT was estimated according to Matsuda and DeFronzo [29].

### Intravenous glucose tolerance test (IVGTT) and hyperinsulinemic-euglycemic clamp

In a subgroup of informed subjects (n = 320), an IVGTT was performed after a 10-h overnight fast, as described earlier [30]. After baseline samples had been collected (−10, −5, and 0 min), a glucose dose of 0.3 g/kg body weight was given at time point 0. Blood samples for the measurement of plasma glucose and insulin were obtained at 2, 4, 6, 8, and 10 minutes. In these subjects, a hyperinsulinemic-euglycemic clamp for the determination of insulin sensitivity was performed as well [31].

### Analytical procedures

Blood glucose was determined using a bedside glucose analyzer (glucose oxidase method; Yellow Springs Instruments, Yellow Springs, OH, USA). Plasma insulin and proinsulin were determined by microparticle enzyme immunoassay (Abbott Laboratories, Tokyo, Japan, and IBL, Hamburg, Germany, respectively). The proinsulin assay has 0% cross-reactivity with human insulin and C-peptide. The insulin assay has 0% cross-reactivity with proinsulin.

### Calculations and statistical analyses

The area under the curve (AUC) AUC_Ins0–30_/AUC_Glc0–30_ was calculated as (Ins0+Ins 30)/(Glc0+Glc30). Insulinogenic index (IGI) was calculated as (Ins30 – Ins0)/(Glc30 – Glc 0). Cleared insulin response (CIR) was (100×Ins30)/Glc30×(Glc30 – 3.89). AUC was calculated according to the trapezoid method as: 0.5×(C_0_/2+C_30_+C_60_+C_90_+C_120_/2) with c = concentration.

Unless otherwise stated, data are given as mean ± SD. Data that were not normally distributed were logarithmically transformed prior to statistical analysis. A Bonferroni-corrected p-value<0.0127 was considered statistically significant according to the number of SNPs tested.

Hardy-Weinberg equilibrium was tested using χ^2^ test.

Our study was sufficiently powered (1–β>0.8, α = 0.05) to detect effect sizes ≥0.18 for the SNP with the lowest minor allele frequency (rs11085226). Power calculations were performed using G*power software (www.psycho.uni-duesseldorf.de/aap/projects/gpower/). Pairwise linkage disequilibrium (D′, r^2^) was determined using the Java linkage disequilibrium plotter (http://www.genepi.com.au/projects/jlin). For all other statistical analyses, the software package JMP 7.0.1 (SAS Institute, Cary, NC, USA) was used.

### MAGIC genome-wide association (GWA) data

GWA-data on glycaemic traits have been made publicly available by the MAGIC consortium [32] and have been downloaded from www.magicinvestigators.org. We interrogated data from the meta-analysis results for the homeostatic model assessment of β-cell function (HOMA-B).

### Analysis of linkage between polymorphisms

A search for SNPs in linkage disequilibrium with SNP rs123698was performed on the Broad Institute's SNAP(SNP Annotation and Proxy Search) website (http://www.broadinstitute.org/mpg/snap/ldsearch.php) using the CEU panel from both the *1000 genomes project pilot 1* dataset as well as well as *HapMap release 22*.

### Analysis of putative transcription factor binding sites

For these analyses, the JASPAR (http://jaspar.genereg.net) database [33] “homo sapiens” was used with a relative profile score threshold of 80%. We analyzed the 50 bp around each of the two SNPs that showed associations with insulin levels during IVGTT and all linked SNPs with r^2^>0.8 (1000 genomes project pilot 1: SNPs rs10420407 and rs10420953 for SNP rs11085226; SNPs rs351976, rs8103323, rs8100561, and rs351995 for SNP rs351974).

## Results

### Subject characteristics

The participants' clinical characteristics are presented in Table 1. In this group of relatively young subjects 66% were females and 34% males. They were slightly overweight and non-diabetic. 10% of the subjects had impaired fasting glucose, 10% had impaired glucose tolerance and 7% had both.

### Genotyping

We analyzed the following tagging SNPs: rs351974, rs11085226, rs736926, and rs123698 in *PTBP1*. All SNPs were in Hardy-Weinberg equilibrium (all p≥0.3) and showed MAFs very close to those reported by the HapMap project (http://www.hapmap.org, [28], Table 2).

**Table 2:** Linkage disequilibrium statistics (D′, r^2^) among the four tagging SNPs rs351974, rs11085226, rs736926, and rs123698 covering the 14.92 kb genomic locus harbouring the *PTBP1* gene.

SNP	rs351974	rs11085226	rs736926	rs123698
**rs351974**	-	0.995	0.842	0.996
**rs11085226**	0.238	-	1.000	0.985
**rs736926**	0.279	0.019	-	0.976
**rs123698**	0.254	0.071	0.096	-
**MAF (MAF_HapMap_)**	0.293 (0.292)	0.106 (0.092)	0.140 (0.123)	0.382 (0.405)

D′ values above empty cells; r^2^ values below empty cells. PTBP1–polypyrimidine-tract-binding protein, SNP–single nucleotide polymorphism, MAF–minor allele frequency, MAF_HapMap_–minor allele frequency from theInternationalHapMap Project.

### Insulin secretion during the OGTT

The minor allele of SNP rs11085226 in *PTBP1* was nominally associated with lower insulin secretion measured as IGI and CIR during the OGTT in the dominant inheritance model (p≤0.04; Table 3). Whereas the third insulin-based parameter, i.e., AUC_Ins0–30_/AUC_Glc0–30_ revealed a trend for association, there was no association with C-peptide-based measures (Table 3). The three other tested SNPs in the *PTBP1* gene did not show any association with any of the tested parameters of insulin secretion (all p>0.07; Table 3 and 4). None of the analyzed SNPs was associated with proinsulin levels or proinsulin-to-insulin ratio. In our data, we detected no significant association with HOMA-B.

**Table 3:** Associations of *PTBP1* SNPs rs351974 and rs11085226 with insulin secretion and proinsulin conversion in non-diabetic subjects (N = 1502).

SNP	rs351974					rs11085226				
Genotype	AA	AC	CC	p_1_	p_2_	AA	AG	GG	p_1_	p_2_
**N**	760	604	137	-	-	1205	273	23	-	-
**HOMA-B**	131.2±116.1	136.7±132.1	130.7±126.4	0.8	0.9	132.7±126.2	137.5±116.4	118.6±60.0	0.8	0.8
**AUC_Ins0–30_/AUC_Glc0–30_ (×10^−9^)**	40.8±29.6	40.4±31.7	43.0±31.6	0.7	0.5	41.2±30.7	39.5±29.8	42.0±33.8	0.07	0.08
**IGI (×10^−9^)**	150±299	149±137	147±116	0.9	0.7	153±253	136±113	147±135	0.08	**0.0378**
**CIR (l×mmol^−1^×10^−9^)**	1530±1368	1516±1378	1491±1205	0.9	0.6	1551±1393	1395±1188	1511±1325	0.07	**0.0429**
**C-pep30 (pmol/l)**	2062±880	2019±889	2067±939	1.0	1.0	2055±890	2001±894	2085±783	0.2	0.2
**AUC_C-pep_/AUC_Glc_ (×10^−9^)**	320±103	318±108	317±108	0.8	0.9	320±103	316±115	332±100	0.2	0.2
**Proins0 (pmol/l)**	5.80±6.67	5.92±6.86	4.91±4.14	0.5	0.7	5.78±6.83	5.81±5.44	5.00±3.71	0.3	0.1
**Proins30 (pmol/l)**	11.6±9.6	12.0±15.5	11.8±8.4	0.7	0.8	11.7±12.8	12.0±9.9	12.1±8.6	0.8	0.6
**AUC_Proins_ (pmol/l)**	35.4±27.7	36.9±38.5	37.6±28.7	0.8	0.9	35.7±31.3	38.5±37.8	37.3±26.5	0.9	0.7
**Proins0/Ins0**	0.124±0.173	0.136±0.234	0.112±0.156	0.6	0.8	0.128±0.207	0.128±0.162	0.120±0.137	0.4	0.2
**Proins30/Ins30**	0.032±0.033	0.033±0.054	0.049±0.035	0.8	0.5	0.032±0.043	0.034±0.041	0.033±0.031	0.4	0.2
**AUC_Proins_/AUC_Ins_**	0.049±0.041	0.052±0.059	0.049±0.035	0.9	1.0	0.050±0.050	0.052±0.043	0.056±0.050	0.8	0.5

Data represent means ±SD. For statistical analysis, data were ln-transformed and adjusted for gender, age, BMI, and insulin sensitivity. p_1_ – p-values for the additive inheritance model; p_2_ – p-values for the dominant inheritance model. AUC – area under the curve; BMI – body mass index; CIR – cleared insulin response; IGI – insulinogenic index; SNP – single nucleotide polymorphism.

**Table 4:** Associations of *PTBP1* SNPs rs736926 and rs123698 with insulin secretion and proinsulin conversion in non-diabetic subjects (N = 1502).

SNP	rs736926					rs123698				
Genotype	CC	CT	TT	p_1_	p_2_	CC	CG	GG	p_1_	p_2_
**N**	1112	357	30	-	-	577	692	224	-	-
**HOMA-B**	132.1±126.1	135.2±115.9	157.8±127.9	0.2	0.5	133.8±123.3	135.3±130.5	128.0±103.6	0.8	0.4
**AUC_Ins0–30_/AUC_Glc0–30_ (×10^−9^)**	40.6±30.3	40.9±31.5	47.7±30.8	0.3	0.5	39.8±29.4	42.4±32.6	38.7±27.5	0.2	0.2
**IGI (×10^−9^)**	147±257	157±141	164±115	0.2	0.1	138±147	168±252	121±330	0.07	0.1
**CIR (l×mmol^−1^×10^−9^)**	1521±1406	1501±1198	1678±1319	0.3	0.3	1443±1231	1610±1476	1452±1288	0.1	0.2
**C-pep30 (pmol/l)**	2050±880	2008±891	2280±1178	0.7	0.6	2011±864	2080±908	2024±894	0.4	0.2
**AUC_C-pep_/AUC_Glc_ (×10^−9^)**	320±106	313±103	344±122	0.4	0.6	316±105	322±107	318±102	0.6	0.3
**Proins0 (pmol/l)**	5.82±6.55	5.51±6.68	6.70±5.66	0.09	0.3	5.79±7.30	5.76±6.08	5.81±6.02	0.4	0.2
**Proins30 (pmol/l)**	11.8±9.9	11.6±17.6	14.2±11.7	0.3	0.3	11.8±15.8	11.9±9.8	11.3±7.9	0.5	0.2
**AUC_Proins_ (pmol/l)**	36.0±30.5	36.1±38.2	42.1±28.2	0.7	1.0	37.5±37.1	35.4±30.7	35.1±23.9	0.5	0.8
**Proins0/Ins0**	0.125±0.167	0.137±0.277	0.141±0.150	0.1	0.2	0.131±0.244	0.124±0.169	0.132±0.149	0.3	0.4
**Proins30/Ins30**	0.032 0.035	0.033±0.061	0.031±0.024	0.4	0.2	0.033±0.055	0.032±0.031	0.033±0.034	0.6	0.5
**AUC_Proins_/AUC_Ins_**	0.049±0.041	0.053±0.068	0.051±0.038	0.9	1.0	0.052±0.060	0.049±0.040	0.051±0.038	0.9	1.0

Data represent means ±SD. For statistical analysis, data were ln-transformed and adjusted for gender, age, BMI, and insulin sensitivity. p_1_ – p-values for the additive inheritance model; p_2_ – p-values for the dominant inheritance model. AUC – area under the curve; BMI – body mass index; CIR – cleared insulin response; IGI – insulinogenic index; SNP – single nucleotide polymorphism.

### Insulin secretion during the IVGTT

**Figure 2:**
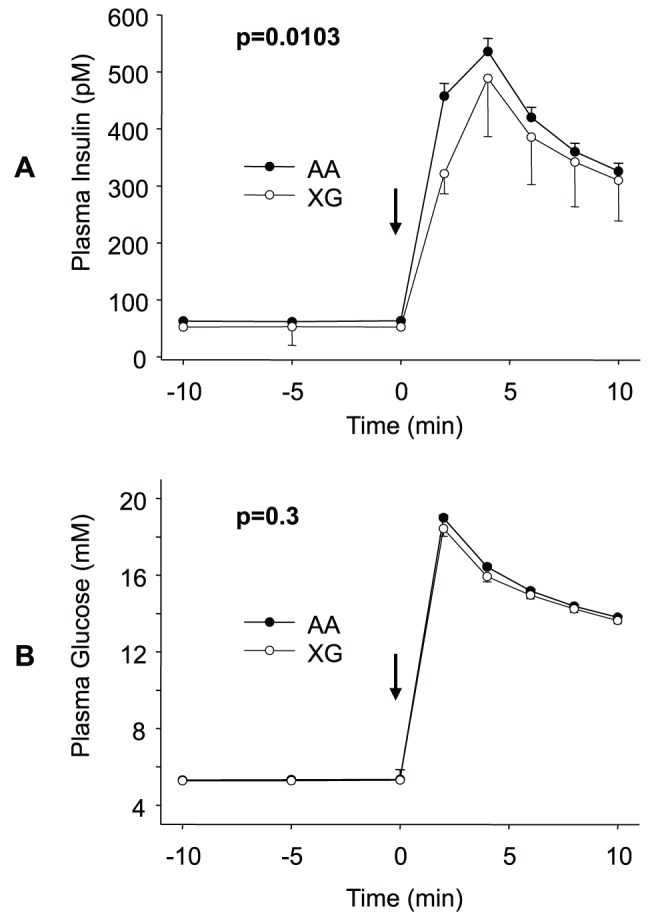
Metabolic data in carriers of the PTBP1 SNP rs11085226 during the IVGTT. Shown are plasma insulin (A) and glucose (B) concentrations. Data are given as means +/− SEM. P-values for AUC of the ten minutes following glucose injection adjusted for gender, age, BMI, and clamp-derived insulin sensitivity are given. Arrows mark the time-point of glucose infusion. Black circles: homozygous carriers of the major allele; white circles: heterozygous and homozygous carrier of the minor allele.

The minor allele of SNP rs11085226 showed significant association with lower insulin levels within the first ten minutes following glucose injection (AUC_0–10 min_ p = 0.0103, dominant inheritance model; Figure 2 A), while glucose levels did not diverge (AUC_0–10 min_ p = 0.3; Figure 2 B).

Neither SNP rs736926, nor SNP rs123698 were associated with insulin levels during the IVGTT (AUC_0–10 min_ p = 0.4 and p = 0.7, respectively), in a model adjusted for gender, age, BMI, and clamp-derived insulin sensitivity index. Performing the same adjustment, SNP rs351974 was associated with insulin levels during the first 10 minutes of the IVGTT (AUC_Insulin 0–10 min_ p = 0.0108, dominant inheritance model). SNP rs351974 was furthermore associated with glucose levels during the same time period after adjustment for gender, age, and BMI (AUC_Glucose 0–10 min_ p = 0.0172, dominant inheritance model). Both, glucose and insulin levels were lower in carriers of the minor allele of SNP rs351974. The association of this SNP with AUC_Insulin 0–10 min_ remained present after adjustment for AUC_Glucose 0–10 min_ in addition to gender, age, BMI, and clamp-derived insulin sensitivity index (p = 0.0377).

### Replication of the association of SNP rs11085226 with insulin secretion in MAGIC GWA data

In the publicly available data on glycaemic traits from the MAGIC consortium on 46,186 non-diabetic participants [32], SNP rs11085226 was associated with the provided measure of insulin secretion, HOMA-B (p = 0.01815) with higher insulin secretion in carriers of the A-allele. Of the other three investigated *PTBP1* SNPs, rs736926 showed association with HOMA-B in MAGIC, too (p = 0.04164), and SNP rs351974 did not show such an association (p = 0.7). Since SNP rs123698 is not included in the provided MAGIC data set, we searched for linked polymorphisms (r^2^>0.8)in 1000 genomes pilot 1, as well as HapMap release 22 but found no such linked SNPs. Since we were unable to identify another polymorphism as a proxy for *PTBP1*rs123698, we could not check for associations between this SNP and HOMA-B in MAGIC data.

## Discussion

In the present study, we found that the common variant rs11085226 in the *PTBP1* gene is associated with significantly lower glucose-stimulated insulin secretion, but not with altered proinsulin-to-insulin ratio. This finding could be replicated in the huge data set from the MAGIC consortium. We furthermore found SNP rs351974 to be associated with insulin levels during the IVGTT, but not with any of the OGTT-derived parameters in our study, or in the analyzed MAGIC data.

The influence of *PTBP1* rs11085226 in the IVGTT especially on the insulin levels within few minutes after glucose injection with diminished influence at later time points might explain why we could only detect effects on OGTT-derived parameters of insulin secretion that cover the first 30 minutes. The large sample size of MAGIC [32] was necessary to detect also effects of this SNP on fasting insulin secretion (HOMA-B), most likely because glucose-stimulated insulin secretion plays only a minor role in the fasting state. Notably, the effect was not detected in C-peptide-based parameters (C-pep30, AUC_C-pep_/AUC_Glc_). This discrepancy could be due to effects of the SNP on insulin clearance. So, we examined the association of *PTBP1* rs11085226 with two estimates of insulin clearance (Cpep0/Ins0 and AUC_Cpep_/AUC_Ins_). Both parameters were not associated with this SNP (p≥0.3).

The association of *PTBP1* SNP rs351974 with insulin levels (independently of its concomitant association with glucose) was only detectable in the IVGTT data but neither in OGTT data nor in MAGIC. Similar discrepancy between insulin levels after oral and i.v. glucose administration was noted for a polymorphism in *SLC30A8* [34]. The underlying mechanism for this observation still remains open but one possible explanation might be that the incretin effect that is present only after oral glucose uptake compensates for the SNP effects and, thus, making them undetectable during an OGTT.

SNP *PTBP1* rs11085226 is in linkage with two other SNPs, one in intron 3 (rs10420407 [A/G]) and one in exon 5 (rs10420953) (figure 1). The exonic variation is a silent mutation (N108N), thus does not change the amino acid sequence. Therefore, these variations are suggested to influence the expression of *PTBP1* at the transcriptional level rather than altering the protein functions. For instance, they could influence the binding of transcription factors or repressors, however this is unlikely since the JASPAR database [33] contains no putative transcription factor binding sites around the SNPs or linked SNPs. Furthermore, the silent mutation in exon 5 could also affect RNA processing or protein translation rate. SNP rs11085226 could possibly affect alternative splicing, since it is located in an intron next to an alternative splicing site [14], [35].

Since PTBP1 influences the expression of proinsulin convertases, we expected that genetic variations could affect proinsulin/insulin ratios. However, such effects were not found. Interestingly, *PTBP1* SNPs rs11085226 and rs351974 reduced insulin secretion very early after glucose administration but not at later time points. One might speculate therefore that the variation influences the number of insulin secretory granules within the readily releasable pool, which are mainly responsible for the first phase of insulin secretion [36]. Of interest, PTBP1 seems to be involved in impairment of this mechanism in type 2 diabetes [37]. The hypothesis of alterations in the pool of readily releasable granules is supported by the fact that PTBP1 supports the biogenesis of insulin secretory granules, while data in rodent β-cells indicate that newly-synthesized insulin secretory granules undergo preferential exocytosis ([38], [39]; Ivanova and Solimena, unpublished data). Moreover, PTBP1 may regulate the expression of proteins that are directly involved in the machinery for exocytosis of insulin secretory granules.

Our study could only detect an effect size ≥18% and was therefore underpowered to detect smaller effect sizes, e.g. on proinsulin conversion, and thus larger cohorts should be studied to assess such possible smaller effects. Further on, we focused on common variants with a MAF>5%. Therefore we might have overlooked effects of rare variants or mutations that could more dramatically influence PTBP1 levels or functions. Studying such rare variants will need much larger cohorts and direct sequencing strategies. Further clarification about the influence of *PTBP1* SNPs on insulin secretory granule exocytosis will also require additional studies at the molecular level.

Taken together, our data suggest that the common genetic variation in *PTBP1* affects very early glucose-stimulated insulin secretion. Thus, the effects of PTBP1 on insulin secretion that were discovered in model systems are detectable and relevant in humans *in vivo*, too.
